# Simultaneous targeted exchange of two nucleotides by single-stranded oligonucleotides clusters within a region of about fourteen nucleotides

**DOI:** 10.1186/1471-2199-9-14

**Published:** 2008-01-28

**Authors:** Heike Hegele, Matthias Wuepping, Caroline Ref, Oliver Kenner, Dieter Kaufmann

**Affiliations:** 1 Institute of Human Genetics, University of Ulm, D 89070 Ulm, Germany

## Abstract

**Background:**

Transfection of cells with gene-specific, single-stranded oligonucleotides can induce the targeted exchange of one or two nucleotides in the targeted gene. To characterize the features of the DNA-repair mechanisms involved, we examined the maximal distance for the simultaneous exchange of two nucleotides by a single-stranded oligonucleotide. The chosen experimental system was the correction of a *hprt-*point mutation in a hamster cell line, the generation of an additional nucleotide exchange at a variable distance from the first exchange position and the investigation of the rate of simultaneous nucleotide exchanges.

**Results:**

The smaller the distance between the two exchange positions, the higher was the probability of a simultaneous exchange. The detected simultaneous nucleotide exchanges were found to cluster in a region of about fourteen nucleotides upstream and downstream from the first exchange position.

**Conclusion:**

We suggest that the mechanism involved in the repair of the targeted DNA strand utilizes only a short sequence of the single-stranded oligonucleotide, which may be physically incorporated into the DNA or be used as a matrix for a repair process.

## Background

Transfection of cells with single-stranded oligonucleotides showing a mismatch to a target gene sequence can result in an exchange of the single nucleotide in the genomic DNA [1-8]. The repair mechanisms involved in this targeted gene alteration (TGA) are still under discussion [9-12]. Previous findings indicate that in an initial step single-stranded oligonucleotides anneal to the targeted strand of the gene and that *RAD51*, *RAD54 *and *XRCC2 *are involved in this process [13-15]. In a second step the repair of the targeted strand takes place and the oligonucleotide is either physically incorporated into the target DNA [8,16-18] or serves as a matrix for specific repair mechanisms. Proteins involved in mismatch repair (MMR) seem to be crucial for this nucleotide exchange in yeast but not in mammalian cells [19]. Another possible repair mechanism involved in this step is nucleotide exchange repair [20]. The participation of double-strand break repair and homologous recombination has also been suggested [8,21-26]. The alteration of the sequence of the target strand results in a new mismatch between the two strands of DNA helix. In a third step the repair of emerged mismatches between the corrected targeted strand and its complementary strand via different repair pathways takes place, thus generating an intact DNA helix [4].

In the present study we sought to characterize one feature of the mechanisms involved in the targeted gene alteration, namely the extent of the sequence of the single-stranded oligonucleotide which is used for the correction of the targeted gene.

One method to examine this is the transfection of oligonucleotides carrying at least two specific markers and the detection of their simultaneous appearance in the target DNA. In our study we used two mismatches of the oligonucleotides to the target sequence as markers. The first marker is a nucleotide which alters the premature stop codon (TGA) in the *hprt *deficient V79-151 cells to a codon for Arginine (*hprt *position 151, CGA) or for Cysteine (*hprt *position 153, TGC) (Table 1). Both exchanges restore *hprt *function and thereby allow the selection of cells by incubation in HAT medium [2]. The second marker is a nucleotide the exchange of which leads to a silent mutation in *hprt *(Table 1). The simultaneous exchange of two nucleotides in *hprt *by transfection with single-stranded oligonucleotides carrying two mismatches has been demonstrated by us before [27] and in an episomal yeast target gene by Agarwal et al. [28]. Agarwal et al. carried out *in vitro *experiments with oligonucleotides carrying two mismatches to an episomal target plasmid. The first mismatch directed the repair of a hygromycin point mutation and the second generated a silent mutation which leads to a new restriction enzyme cleavage site. A similar approach was used to examine the physical incorporation of transfected oligonucleotides into the DNA [18]. Here, the correction of a point mutation and the simultaneous occurrence of a biotin labeled nucleotide were used as markers. We suggest that if DNA sequence analysis of transfected cells shows the exchange of both targeted nucleotides, at least the region of the oligonucleotide situated between the two mismatches has been used for TNE.

**Table 1:** *Hprt *sequences

**Sequences of coding strand of *hprt *intron 2 and exon 3 in V79 wildtype and V79-151 cells**.		
**V79 wildtype**	**position 151**	

DNA sequence	5'-ttgtag G ACT GAA AGA CTT GCC ***C***GA GAT GTC ATG AAA GAG ATG GGA-3	
amino acids	Thr Glu Arg Leu Ala **Arg **Asp Val Met Lys Glu Met Gly	

**V79-151**	**position 151**	

DNA sequence	5'-ttgtag G ACT GAA AGA CTT GCC ***T***GA GAT GTC ATG AAA GAG ATG GGA-3	
amino acids	Thr Glu Arg Leu Ala **Stop **Asp Val Met Lys Glu Met Gly	

**Positions of the intended first nucleotide exchanges for correcting the *hprt *mutation**.		

	**Exchange of nucleotide on position 151**	

DNA sequence	5'-ttgtag G ACT GAA AGC CTT GCC ***C***GA GAT GTT ATG AAA GAG ATG GGA-3'	
amino acids	Thr Glu Arg Leu Ala **Arg **Asp Val Met Lys Glu Met Gly	

	**Exchange of nucleotide on position 153**	

DNA sequence	5'-ttgtag G ACT GAA AGC CTT GCC TG***C ***GAT GTT ATG AAA GAG ATG GGA-3'	
amino acids	Thr Glu Arg Leu Ala **Cys **Asp Val Met Lys Glu Met Gly	

**Positions of the intended second nucleotide exchange generating a silent mutation**		

**Position**	**Base exchange**	**Sequence of coding strand of hprt intron 2 in V79-151 cells**.

141	A to G	5'-ttgtag G ACT GA**G **AGA CTT GCC *T*GA GAT GTC ATG AAA GAG ATG GGA-3
144	A to G	5'-ttgtag G ACT GAA AG**G **CTT GCC *T*GA GAT GTC ATG AAA GAG ATG GGA-3
147	T to C	5'-ttgtag G ACT GAA AGA CT**C **GCC *T*GA GAT GTC ATG AAA GAG ATG GGA-3
150	C to T	5'-ttgtag G ACT GAA AGA CTT GC**T ***T*GA GAT GTC ATG AAA GAG ATG GGA-3
156	T to C	5'-ttgtag G ACT GAA AGA CTT GCC *T*GA GA**C **GTC ATG AAA GAG ATG GGA-3
159	C to T	5'-ttgtag G ACT GAA AGA CTT GCC *T*GA GAT GT**T **ATG AAA GAG ATG GGA-3
165	A to G	5'-ttgtag G ACT GAA AGA CTT GCC *T*GA GAT GTC ATG AA**G **GAG ATG GGA-3
168	G to A	5'-ttgtag G ACT GAA AGA CTT GCC *T*GA GAT GTC ATG AAA GA**A **ATG GGA-3

The table shows the sequence of coding strand of *hprt *intron 2 (lower case letters) and exon 3 (upper case letters) in V79 wildtype cells and V79-151 cells, which carry a stop codon at position 151. Also shown is the sequence of coding strand of *hprt *after base exchange at position 151 or 153 and after base exchange at the second mismatch position, which generates a silent mutation. The exchanged bases are indicated in bold. The respective second base exchanges are generated through the following oligonucleotides: position 141: oligonucleotide H7-151-141; position 144: H5-151-144; position 147: H3-151-147, O45-151-147, C153-147; position 150: H2-151-150, O45-151-150, C153-150; position 156: H4-151-156, O42-151-156, C153-156; position 159: H6-151-159, O25-151-159, O30-151-159, O45-151-159, O48-151-159, C153-159; position 165: H8-151-165; position 168: H9-151-168.

There are several preconditions to our approach. First, the observed exchanges have to be located in *cis *on the same allele of the gene. As *hprt *is an X-chromosomal gene and the cell line we used derives from a male hamster (V79-151), each cell carries only one *hprt *allele and therefore no additional exchange position exists. A second prerequisite is that the exchange of both nucleotides takes place simultaneously. In our cellular system, 10^4 ^cells were transfected per well and the correction rate of about four clones per 10^6 ^transfected cells is very low [2]. It is therefore quite unlikely that two independently targeted nucleotide exchanges occur in one well and, more importantly, in one cell. The next prerequisite for our approach is the intracellular uptake of oligonucleotides which was surveyed through transfection with fluorescently labeled oligonucleotides. Furthermore, it is possible that the intracellular integrity of the oligonucleotides is affected by the activity of exonucleases which accelerate the degradation of oligonucleotides [18]. This is important for our experiments because it might influence the observed nucleotide exchange rate. We therefore determined the minimal distance between the second exchange nucleotide and the 3'-end of the oligonucleotide idem. Finally, the distance between the two mismatched nucleotides should not affect the affinity of the oligonucleotides to the target sequence. This can be monitored through differences in correction rates of the selectable *hprt *mutation. Also, the specific sequence environment of the first exchange nucleotide may influence this kind of experiment. This can be tested by comparing experiments using oligonucleotides correcting the *hprt *mutation at different positions.

Our investigations showed that the smaller the distance between the two mismatches, the higher the probability of a simultaneous exchange became. The simultaneous exchanges were found to be clustered in a region of about fourteen nucleotides upstream and downstream of the first exchange position.

## Results

### Investigation of the minimal distance of the second mismatch nucleotide from the 3'-end of the oligonucleotides

**Figure 1:**
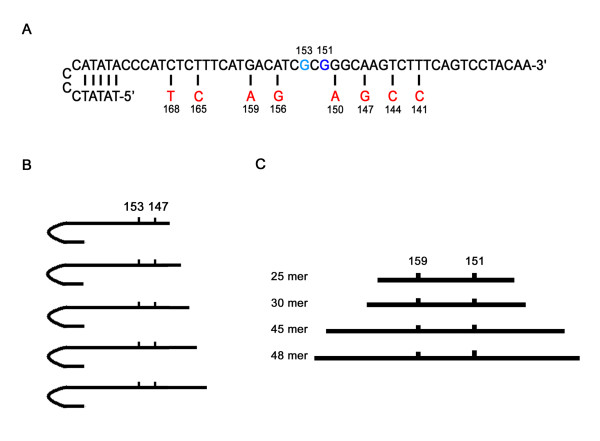
**Structure of the oligonucleotides and relative position of the two mismatches**. (A) Sequence and structure of the oligonucleotides modified with a TA-clamp generating a first nucleotide exchange at position 153 (light blue) ore 151 (dark blue) respectively. The possible positions for the second (silent) exchange are given in red. (B) Structure of the oligonucleotides showing the first mismatch at position 153 and the second mismatch at position 147 in various distance from the unmodified 3'-end. (C) Structure of the oligonucleotides with different length, modified with PTO and showing the first mismatch at position 151 and the second mismatch at position 159.

**Figure 2:**
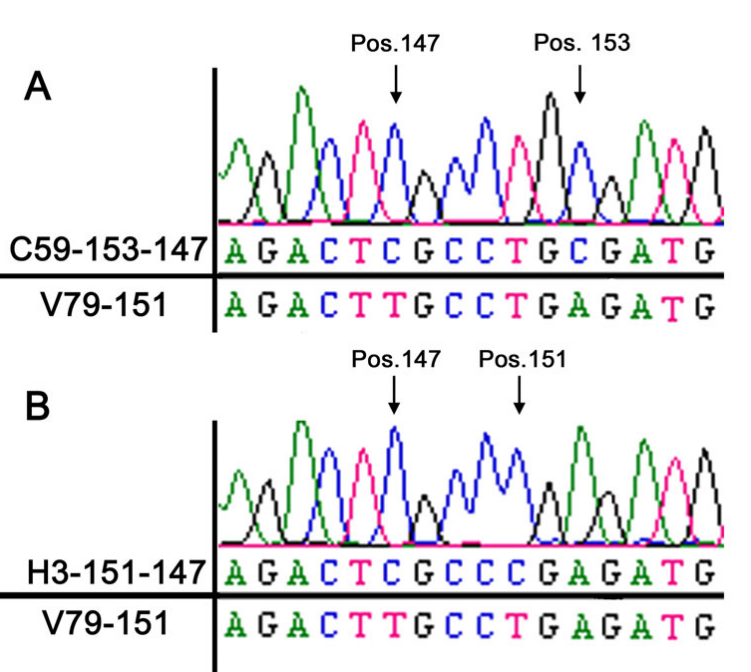
**Sequence analyses of *hprt***. Sequence analysis of the *hprt *gene isolated from V79-151 cell clones after transfection with oligonucleotide C59-153-147 (A) and H3-151-147 (B). Arrows point to the position of the successfully exchanged nucleotides. The upper base code shows the sequence of the analyzed clones, the lower base code the original sequence of the transfected V79-151 cells.

The oligonucleotides used were delivered into the cell by magnet-assisted transfection. The cellular uptake was tested by transfection with 3'-end FITC labeled 5'-TA-clamp oligonucleotides. After incubation for one hour, fluorescence microscopy revealed an uptake of the labeled oligonucleotides into the nucleus in 80 per cent of V79-151 cells. Recently, we observed a rapid degradation of these oligonucleotides in isolated cytoplasm and nucleoplasm by exonuclease and endonuclease activities (unpublished data, M Wuepping). As a result of intracellular degradation through exonucleases the second marker nucleotide lying closer to the 3'- or 5'-end of the oligonucleotide could be removed faster than the first nucleotide positioned in the middle of the sequence and therefore not be exchanged. Thus the rate of simultaneous nucleotide exchange might be influenced by the distance of the second mismatch from the 3'- or 5'-end of the oligonucleotide. To test this hypothesis, cells were transfected with 5'-TA clamp modified oligonucleotides showing the first mismatch at position 153 at a distance of twenty nucleotides to the TA-clamp (Fig. 1A). Using these oligonucleotides the stop codon (*T*GA) of the V79-151 cells is replaced by a codon for cysteine (TG*C*, position 153). The second mismatch resulting in a silent mutation is located at position 147 at a distance of six, twelve, eighteen, twenty-four and thirty nucleotides from the unmodified 3'-end (Fig. 2B, Table 2). The reduction of the exchange rate of the second mismatch nucleotide should indicate the interfering effect of 3'-exonucleases. After about two weeks HAT resistant clones could be found in all experiments (Additional file 1) and DNA sequencing showed the correction at *hprt *position 153 in all sequenced clones. In several clones an additional exchange at position 147 was detected (Fig. 2A). The relative nucleotide exchange rate at position 147 and the number of clones varied depending on the distance of the second mismatch nucleotide from the unmodified 3'-end (Table 3). The total number of clones per 10^6 ^transfected cells increased with decreasing distance of the second mismatch from the 3'-end. However, we found a reduction in the exchange rate of the second nucleotide using oligonucleotides with their second mismatch at a distance of six nucleotides from the 3'-end. Therefore, the 5'-TA-clamp oligonucleotides used in the following experiments carried their second mismatch nucleotide at a distance of twelve nucleotides or more from the unmodified 3'-end.

**Table 2:** Sequences of single-stranded oligonucleotides binding at the *hprt *coding strand

**Oligonucleotide**	**Sequence**
**Oligonucleotides with a first mismatch at position 153 and a second mismatch at position 147 in different distances to the 3'-end**	

C47-153-147	5'-*TATATCCCCATATA*CCCATCTCTTTCATGACATC**G**CAGGC**G**AGTCTT-3'
C53-153-147	5'-*TATATCCCCATATA*CCCATCTCTTTCATGACATC**G**CAGGC**G**AGTCTTTCAGTC-3'
C59-153-147	5'-*TATATCCCCATATA*CCCATCTCTTTCATGACATC**G**CAGGC**G**AGTCTTTCAGTCCTACAA-3'
C65-153-147	5'-*TATATCCCCATATA*CCCATCTCTTTCATGACATC**G**CAGGC**G**AGTCTTTCAGTCCTACAAAAATAG-3'
C71-153-147	5'-*TATATCCCCATATA*CCCATCTCTTTCATGACATC**G**CAGGC**G**AGTCTTTCAGTCCTACAAAAATAGAATCAC-3'

**Oligonucleotides with a first mismatch at position 151 and a second mismatch in various distance to the first**	

H1-151	5'-*TATATCCCCATATA*CCCATCTCTTTCATGACATCTC**G**GGCAAGTCTTTCAGTCCTACAA-3'
H7-151-141	5'-*TATATCCCCATATA*CCCATCTCTTTCATGACATCTC**G**GGCAAGTCT**C**TCAGTCCTACAA-3'
H5-151-144	5'-*TATATCCCCATATA*CCCATCTCTTTCATGACATCTC**G**GGCAAG**C**CTTTCAGTCCTACAA-3'
H3-151-147	5'-*TATATCCCCATATA*CCCATCTCTTTCATGACATCTC**G**GGC**G**AGTCTTTCAGTCCTACAA-3'
H2-151-150	5'-*TATATCCCCATATA*CCCATCTCTTTCATGACATCTC**GA**GCAAGTCTTTCAGTCCTACAA-3'
H4-151-156	5'-*TATATCCCCATATA*CCCATCTCTTTCATGAC**G**TCTC**G**GGCAAGTCTTTCAGTCCTACAA-3'
H6-151-159	5'-*TATATCCCCATATA*CCCATCTCTTTCAT**A**ACATCTC**G**GGCAAGTCTTTCAGTCCTACAA-3'
H8-151-165	5'-*TATATCCCCATATA*CCCATCTC**C**TTCATGACATCTC**G**GGCAAGTCTTTCAGTCCTACAA-3'
H9-151-168	5'-*TATATCCCCATATA*CCCAT**T**TCTTTCATGACATCTC**G**GGCAAGTCTTTCAGTCCTACAA-3'

**Oligonucleotides with a first mismatch at position 153 and a second mismatch in various distance to the first**	

H153-1T	5'-*TATATCCCCATATA*CCCATCTCTTTCATGACATC**G**CAGGCAAGTCTTTCAGTCCTACAA-3'
C153-147	5'-*TATATCCCCATATA*CCCATCTCTTTCATGACATC**G**CAGGC**G**AGTCTTTCAGTCCTACAA-3'
C153-150	5'-*TATATCCCCATATA*CCCATCTCTTTCATGACATC**G**CA**A**GCAAGTCTTTCAGTCCTACAA-3'
C153-156	5'-*TATATCCCCATATA*CCCATCTCTTTCATGAC**G**TC**G**CAGGCAAGTCTTTCAGTCCTACAA-3'
C153-159	5'-*TATATCCCCATATA*CCCATCTCTTTCAT**A**ACATC**G**CAGGCAAGTCTTTCAGTCCTACAA-3'

**Oligonucleotides with a first mismatch at position 151 and a second mismatch in various distance to the first**	

O45-151-147	5'-C*C*C*A*TCTCTTTCATGACATCTC**G**GGC**G**AGTCTTTCAGTCCT*A*C*A*A-3'
O45-151-150	5'-C*C*C*A*TCTCTTTCATGACATCTC**GA**GCAAGTCTTTCAGTCCT*A*C*A*A-3'
O42-151-156	5'-T*C*C*C*ATCTCTTTCATGAC**G**TCTC**G**GGCAAGTCTTTCAG*T*C*C*T-3'
O45-151-159	5'-C*C*C*A*TCTCTTTCAT**A**ACATCTC**G**GGCAAGTCTTTCAGTCCT*A*C*A*A-3'

**Oligonucleotides in different length with a first mismatch at position 151 and a second mismatch at position 159**	

O-25-151C-159T	5'-T*T*T*C*AT**A**ACATCTC**G**GGCAAG*T*C*T*T-3'
O-30-151C-159T	5'-C*T*C*T*T TCAT**A**ACATCTC**G**GGCAAGTC*T*T*T*C-3'
O-45-151C-159T	5'-C*C*C*A*TCTCTTTCAT**A**ACATCTC**G**GGCAAGTCTTTCAGTCCT*A*C*A*A-3'
O-48-151C-159T	5'-C*C*T*C*CCATCTCTTTCAT**A**ACATCTC**G**GGCAAGTCTTTCAGTCCT*A*C*A*A-3'

The 5' TA-clamps are indicated in italics and the phosphorothioates are indicated by asterisks. The positions of the first and second mismatch are indicated in bold and underlined.

**Table 3:** Percentage of clones with both exchanges obtained with different oligonucleotides

**Positions 153 and 147 (in different distances to the 3'-end)**			
**Oligonucleotide**	**Distance of position 147 to the 3'-end (number of nucleotides)**	**number of clones per 10**^6^**transfected cells**	**% of clones with both exchanges**

C47-153-147	6	22	16
C53-153-147	12	13,3	54
C59-153-147	18	9,7	63
C65-153-147	24	8,7	47
C71-153-147	30	6,3	62

**Positions 151 + x (TA-clamps)**			

**Oligonucleotide**	**Distance (nucleotides) to the first position**	**number of clones per 10**^6^**transfected cells**	**% of clones with both exchanges**

H1-151	-	4.86	-
H7-151-141	-10	4.00	33
H5-151-144	-7	1.67	50
H3-151-147	-4	1.83	91
H2-151-150	-1	1.33	100
H4-151-156	5	2.67	63
H6-151-159	8	1.83	27
H8-151-165	14	4.00	8
H9-151-168	17	6.00	0

**Positions 153 + x (TA-clamps)**			

**Oligonucleotide**	**Distance (nucleotides) to the first position**	**number of clones per 10**^6^**transfected cells**	**% of clones with both exchanges**

H153-1T	-	18.00	-
C-153-147	-6	4.67	50
C-153-150	-3	1.00	100
C-153-156	3	2.67	89
C-153-159	6	5.00	35

**Positions 151 + x (PTO modifications)**			

**Oligonucleotide**	**Distance (nucleotides) to the first position**	**number of clones per 10**^6^**transfected cells**	**% of clones with both exchanges**

O45-151-147	-4	3.50	64
O45-151-150	-1	0.33	100
O42-151-156	5	6.50	41
O45-151-159	8	1.33	8

**Positions 151 + 159, different lengths (PTO modifications)**			

**Oligonucleotide**	**Length of the oligonucleotides (nucleotides)**	**number of clones per 10**^6^**transfected cells**	**% of clones with both exchanges**

O25-151-159	25	2.83	12
O30-151-159	30	3.83	13
O45-151-159	45	1.33	13
O48-151-159	48	2.00	17

The number of HAT resistant clones per 10^6 ^transfected cells and the percentage of clones showing the simultaneous exchange of both mismatches is given depending on the distance of the second mismatch nucleotide to the 3'-end of the oligonucleotide, the distance of the second mismatch to the first position or length of the oligonucleotides.

### Nucleotide exchange at *hprt *position 151 and simultaneous exchange of an additional nucleotide at various distances

**Figure 3:**
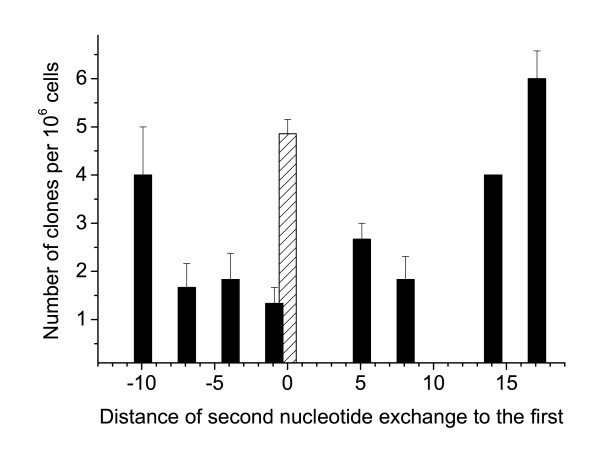
**Correction rate of the first mismatch position**. Correction rate of the first mismatch position after transfection with TA-clamp modified oligonucleotides carrying one mismatch (striped column) or two mismatches (black columns). The first exchange nucleotide was at *hprt *position 151 and the second mismatch at various distances. The X-axis indicates the distance of the second mismatch from the first exchange position in upstream and downstream direction. The Y-axis shows the mean number of clones obtained per 10^6 ^transfected cells. Mean and standard deviations were calculated based on the total number of clones after 3 – 6 transfection experiments per oligonucleotide (Additional file 1).

Next, the simultaneous exchange of two nucleotides at different positions was investigated. We transfected the V79-151 cells with 5'-TA-clamp oligonucleotides showing a first mismatch to the targeted mutated *hprt *sequence at position 151 and a second at position 141/144/147/150/156/159/165 or 168, respectively (Fig. 1A; Tables 1 and 2). After about two weeks one to seven HAT resistant clones per 10^6 ^transfected cells could be found in each transfection experiment (Additional file 1). After transfection with a control oligonucleotide carrying only one mismatch to the target sequence we obtained on average 4.9 clones per 10^6 ^transfected cells. The insertion of an additional mismatch to the target sequence at a distance of one to eight nucleotides from the first mismatch reduced the number of clones by about fifty percent (Fig. 3). This reduction might be due to a reduced affinity of the oligonucleotides with two mismatches to the target sequence. As the number of clones was almost the same among all of these oligonucleotides we assume that they all exhibit a comparable affinity to the target strand. Interestingly, in experiments with oligonucleotides carrying the second mismatch at a distance of ten to seventeen nucleotides from the first mismatch the number of clones received was comparable to experiments using the oligonucleotide carrying only one mismatch.

**Figure 4:**
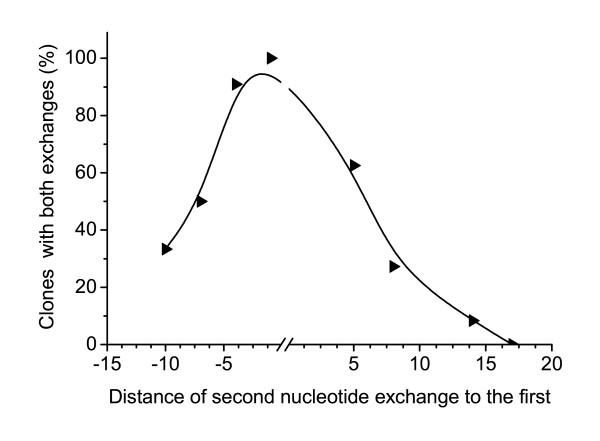
**Exchange rate of the second nucleotide**. Percentage of clones with both exchanges after transfection with TA-clamp modified oligonucleotides carrying their first exchange nucleotide at *hprt *position 151. The X-axis indicates the distance of the second mismatch from the first exchange position in upstream and downstream direction.

DNA sequencing of each HAT resistant clone showed the correction of the point mutation at *hprt *position 151. In several clones the additional intended exchanges at positions 141 to 168 were found (Fig. 2B). The number of clones showing both exchanges varied depending on the distance of the second exchange position to the first exchange at *hprt *position 151 (Table 3, Fig. 4). The smaller the distance between the two exchange positions the higher the rate of simultaneous exchanges was.

### Simultaneous nucleotide exchange at *hprt *position 153 and additional positions in various distances

The specific sequence environment of the first exchange nucleotide may influence this kind of experiment. Therefore we repeated these experiments using oligonucleotides correcting the *hprt *mutation at *hprt *position 153 (Fig. 1A, Table 2). The second nucleotide exchange generated by these oligonucleotides was intended at *hprt *positions 147, 150, 156 and 159. DNA sequencing showed an exchange of the first mismatch at position 153 in all of the received clones. The number of clones after transfection with the control oligonucleotide correcting only position 153 (H153-1T) was higher than using the control oligonucleotide correcting only position 151 (H1-151) (18 clones/10^6 ^cells versus 4.9/10^6^) (Additional file 1). The differences between the two oligonucleotides may be related to the specific sequence environment resulting in different affinities to the target sequence. In the experiments with oligonucleotides carrying two mismatches, the average number of HAT resistant clones was about 3.5 clones per 10^6 ^transfected cells and therefore also higher than in the corresponding experiments exchanging the first nucleotide at position 151 (Additional file 1).

Again, we observed that the exchange rate of the second nucleotide depends on its distance to the first exchange position. We found that the longer the distance between the two mismatches, the lower the rate of clones showing both nucleotide exchanges is (Table 3). This suggests that the variation in the rate of simultaneously exchanged nucleotides is not due to the specific sequence environment.

### Influence of PTO modifications of the oligonucleotides on the exchange rates

The question arises as to whether the type of 3'- and 5'-end modification of the oligonucleotides has an influence on the variation in the number of clones with two nucleotide exchanges. We therefore additionally tested oligonucleotides with PTO modifications at the 3'- and 5'-end which we expected to exhibit a better protection against degradation through exonucleases. These oligonucleotides generate a first exchange at position 151 and a second at the positions 147, 150, 156 or 159 (Table 2). As in the experiments with oligonucleotides modified by 5'-TA clamps, the longer the distance between the two mismatches of the PTO modified oligonucleotides, the lower the rate of two simultaneously exchanged nucleotides was (Table 3). This suggests that this modification of the oligonucleotides has no obvious influence on the exchange rate of the two nucleotides.

### The length of the oligonucleotide does not influence the exchange rates

In order to test whether the length of the oligonucleotides influences the rate of simultaneous exchange of two nucleotides, cells were transfected with oligonucleotides with lengths of twenty-five, thirty, forty and forty-eight nucleotides, respectively, all carrying mismatches to the target sequence at positions 151 and 159 (Fig. 1C). These oligonucleotides were modified by four PTO at each end (Table 2). The HAT resistant clones we obtained after transfection with these oligonucleotides showed no significant variation in the rates of simultaneous exchange of both nucleotides (Table 3). The length of the oligonucleotides in the investigated range of twenty-five to forty-eight nucleotides therefore has no effect on the exchange rate of two nucleotides.

**Figure 5:**
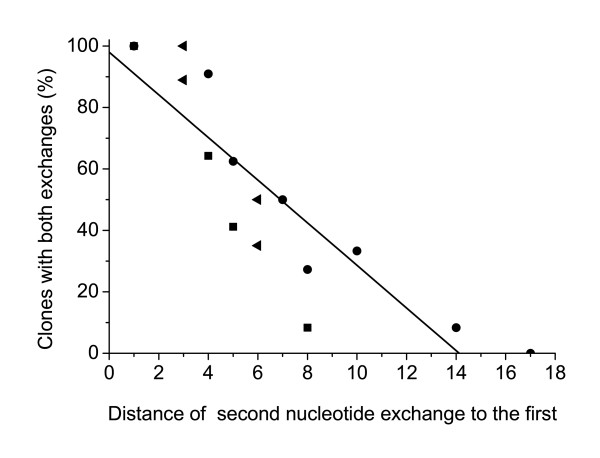
**Fitted exchange rate of the second nucleotide after transfection with differently modified oligonucleotides**. Percentage of clones with both exchanges after different transfection experiments with oligonucleotides carrying two mismatches to *hprt*. The X-axis indicates the distance of the second mismatch (independent of its position in upstream or downstream direction) from the first exchange position. ● first mismatch at position 151, modified by TA-clamps. ◀ : first mismatch at position 153, modified by TA-clamps. ■ : first mismatch at position 151, modified by PTO. The black line shows the linear fit of the data points from the experiments with the three different kinds of oligonucleotides.

Taking all our experimental data together we found that the probability of simultaneous exchange of two nucleotides decreases if the distance between them increases (Fig. 5). We observed a cluster of simultaneously exchanged nucleotides in a *hprt *region of about fourteen nucleotides upstream and downstream of the first exchange position. No more exchanges of the second nucleotide could be observed at a distance of more than fourteen nucleotides. This finding matches with additional experiments where we have not been able to find any exchanges of the second mismatch at a distance of eighteen nucleotides and more (data not shown).

## Discussion and Conclusion

Our experiments on TNE with oligonucleotides carrying two mismatches to the target sequence showed that the longer the distance between the two mismatches, the lower the observed rate of coincident exchanges is. This correlation was found to be independent of the length of the oligonucleotides and two tested terminal modifications protecting against nucleases. The exchange of the first nucleotide at position 153 instead of position 151 led to an increase of the correction efficiency of the first mismatch but had no influence on the rate of the simultaneous exchange of the second nucleotide. We assume that the exchange of both nucleotides takes place during one repair step as the very low correction efficiency makes it quite unlikely that two nucleotides are exchanged independently in one cell. Our data correspond to experiments with oligonucleotides carrying two mismatches using an episomal target in yeast which also indicated that the closer the two sites are, the more likely it is that both will be converted [28]. Other groups found that shifting the mismatch forming nucleotide away from the centre of the targeting vector leads to a decrease in conversion rates [28,29]. Since the oligonucleotides used by these groups had a length of twenty-five nucleotides the reduced correction efficiency may be a consequence of the smaller distance of the mismatch nucleotide to the end of the oligonucleotide. In our experiments defining the minimal distance of the second mismatch nucleotide from the 3'-end of the oligonucleotides we have not been able to observe a higher exchange rate of mismatches lying closer to the centre of the oligonucleotide. But after transfection with oligonucleotides carrying their second mismatch in a distance of only six nucleotides from the 3'-end we found an increase in the correction rate of the first mismatch and a decrease in the correction rate of the second mismatch. We suppose that the reason for this effect is the removal of the second mismatch through a nuclease activity which leads to a higher affinity of the remaining oligonucleotide to the target strand. We have also observed a slight difference in the rate of second exchanges depending on their position in 3'- or 5'-direction relative to the first position. The percentage of exchanged second mismatches was higher when the mismatch was located in 3'-direction from the first mismatch position compared to mismatches located in the same distance in 5'-direction. A corresponding observation has been made by other groups before [29] and they suggested that homology in 5' flanking region is more important for targeted gene alteration than that in 3' flanking region. Since the rates do not differ strongly our observation might be normal variation so we found it not significant enough to draw a conclusion.

One repair mechanism working within a limited sequence region and discussed for the second step of TGA is nucleotide excision repair (NER). The structure-specific endonucleases XPG and ERCC1/XPF are involved in this repair mechanism [30,31] and the participation of these endonucleases in oligonucleotide-directed gene alteration has recently been shown [16]. The limited NER repair region of 22 – 32 nucleotides may be related to our observation of a restricted exchange region. Another repair mechanism working within a limited sequence region is base excision repair (BER) which can be divided into short-patch and long-patch repair. Long-patch repair leads to the exchange of a 2 – 15 nucleotide long DNA fragment [32,33] which would be in line with our findings.

Another repair mechanism that seems to influence TGA is the MMR [34,35]. Especially the MMR protein MSH2 has been shown to suppress the frequency of TGA [19,36]. The influence of specific repair mechanisms on the simultaneous exchange of two nucleotides should be further examined, especially the function of MSH2.

Beside the usage of the oligonucleotide as a matrix for a specific repair mechanism, another possible pathway of TNE is the physical incorporation of the oligonucleotide into the target strand [18]. There are evidences that the incorporation of the oligonucleotide occurs in a replication-dependent way [12,37-41]. The oligonucleotide might serve as an Okazaki fragment and become incorporated into the lagging strand. Okazaki fragments have a length of up to 125 nucleotides [42,43]. Alternatively, the oligonucleotide might serve as a primer for the synthesis of an Okazaki fragment by DNA-Polymerase δ instead of a RNA primer. The length of these primers synthesized by primase is 7–10 ribonucleotides [44].

However, the cluster we observed for the simultaneous exchange of two mismatches of about fourteen nucleotides is shorter than the oligonucleotides used. One possible explanation to fit our findings into the theory of an annealing oligonucleotide which then gets incorporated into the target DNA might be the activity of nucleases. Radecke and colleagues [18] suggest that nucleases might play a role in TGA by rendering transfected oligonucleotides competent for gene correction. It may therefore be hypothesized that the primary oligonucleotide is processed by nucleases to a biologically active shortened molecule. During this process the second mismatch might be removed, depending on its distance to the first. The further apart the two mismatches are, the higher the probability would be that they are separated through a degradation process. We suggest, that the oligonucleotide modifications used for protection against nuclease degradation only work for a very short time but finally also underlie degradation processes.

A observation that supports the hypothesis of a shortened biologically active oligonucleotide is that we found an increased number of clones after transfection with oligonucleotides which carried their second mismatch at a distance of ten and more nucleotides from the first exchange position compared to oligonucleotides with a second mismatch at a distance of less than ten nucleotides from the first exchange position. The loss of the second mismatch after nuclease cleavage might have led to a higher affinity of the shortened oligonucleotide to the target sequence and therefore to a number of clones in the range of oligonucleotides carrying only one mismatch. To better understand the mechanisms underlying TGA further experiments to identify the actual length of the biologically active form of the oligonucleotide should be performed. This could be done by recovering labelled oligonucleotides from corrected cells. Since our experiments showed that transfection of cells with gene-specific, single-stranded oligonucleotides can induce the exchange of nucleotides at various genomic positions the occurance of unexpected exchanges should also be carefully examined. This is especially important for the potential application of TGA in gene therapy.

In summary, we observed that the simultaneous exchange of a second nucleotide clusters within a region of about fourteen nucleotides around the first target nucleotide. We suggest that the biologically active form of the oligonucleotide which is relevant for TNE is only a part of the entire oligonucleotide sequence. The observed limited exchange region might be either the effect of a specific repair mechanism which uses the oligonucleotide as a matrix or due to intercellular emerging degraded oligonucleotides which might also serve as a matrix for the discussed repair mechanisms or be physically incorporated into the target strand.

## Methods

### Cell line and cell culture

A point mutation at position 151 (C → T) of the hprt gene which leads to a stop codon was generated through incubation of V79 cells with ethylmethanesulfonate [2] (Table 1). The V79-151 cells were cultured in DMEM containing ten per cent FBS and maintained at 37°C and five per cent CO_2_. Their doubling time is of about sixteen hours and their G1 phase is therefore very short (six hours). Prior to the transfection experiments, the V79-151 cells were reselected against intact *hprt*-containing cells by incubation with 6-thioguanine (1 μg/ml) for seven days [27].

### Single-stranded oligonucleotides for the correction of the *hprt *mutation and the generation of an additional nucleotide exchange

The single-stranded oligonucleotides (Thermo Electron Corporation, Ulm, Germany)were directed to the non-transcribed strand of *hprt*. They contained one mismatch to the *hprt *sequence of V79-151 cells on cDNA position 151 or 153 (Table 2). For protection against degradation the oligonucleotides were modified either with a TA-clamp at the 5'-end or four phosphorothioates (PTO) on each side. The TA-clamps were constructed on the basis of experiments with the first generation of oligonucleotides for the targeted gene alteration with DNA/RNA-hybrids in which oligonucleotides with symmetric GC-clamps were used [45]. It consists of two complementary sequences of five alternating thymines and adenines which are linked by four cytosines and form a double-stranded loop. The length of the oligonucleotides varied between twenty-five and seventy-one nucleotides and most of the oligonucleotides carried a second mismatch to the *hprt *sequence to generate an additional nucleotide exchange at the positions 141, 144, 147, 150, 156, 159, 165 or 168 (Table 2).

To examine the spontaneous nucleotide exchange rate from T to C at *hprt *position 151 of the V79-151 cells we carried out experiments in which we either transfected them with oligonucleotides not carrying a mismatch to the target sequence or did not treat them with oligonucleotides. The spontaneous nucleotide exchange rate we observed at position 151 was about 0,025 of 10^6 ^cells.

### Transfection of *hprt *(-) V79 cells and selection of hprt (+) cells

*Hprt *(-) V79-151 cells were transfected by a magnet-assisted transfection-system (MATra-A, IBA GmbH, Göttingen, Germany). One day prior to transfection, 10^4 ^cells per well were seeded on a 96-well plate. The oligonucleotides (10 μl, 100 pmol/μl) were incubated with 10 μl MATra-A Reagent and 500 μl serum-free medium at 25°C for fifteen minutes. The solution was then added to 19.6 ml DMEM and 200 μl of the mixture was pipetted into each well. The cells were transfected for sixty minutes on a magnetic plate. Afterwards they were cultured for an additional twenty-four hours in normal culture medium, and selected for three weeks by incubation with HAT medium (InVitrogen Life Technologies, Karlsruhe, Germany). About four hours after the addition of HAT *hprt *deficient V79-151 cells started to die. In some experiments the cells were transfected with polyethylenimine (PEI, ExGen500, Euromedex, Mundolsheim Cedex, France) as described before [27]. The surviving clones were counted and subcloned in HAT medium.

### Investigation of *hprt *sequence alterations in the HAT selected clones

If the number of clones received per transfection experiment was very high, only about fifteen clones per oligonucleotide were sequenced (Additional file 1). The DNA of the HAT selected cell clones was isolated using the QIAmp DNA Blood Mini Kit (Qiagen, Hilden, Germany), quantified by measuring the optical density at 260 nm, and its quality investigated on a 1.5 per cent agarose gel stained with ethidium bromide. The following primer pairs were used for the PCR amplification: 12Hin (5'-TGT GTA AGT ATA ATC TCA GC-3') binding in intron two and 12Rueck (5'-ACA CAG TAG CTC TTC AGT CT-3') binding in exon three; HPRT-Hin5 (5'-TAT TCC GTG ATT TTA TTT TTG TAG-3') binding in intron two and HPRT-Rueck5 (5'-ATG GAT CTA TCA CTA TTT CTA TT-3') binding in exon three. The obtained PCR products have a length of 462 bp and 160 bp respectively. The amplification conditions in a Perkin Elmer GeneAmp 9700 were five minutes at 94°C, thirty-five cycles at 94°C for thirty seconds, 48°C for thirty seconds and 72°C for thirty seconds and a final elongation for seven minutes at 72°C. The PCR products were purified with the Montage PCR Kit (Millipore, Bedford, MA, USA) and sequenced on an ABI 3100 automated sequencer (PE Applied Biosystems, Foster City, CA, USA).

## Abbreviations

Targeted gene alteration (TGA), Nucleotide excision repair (NER), Base excision repair (BER), Mismatch repair (MMR), Phosphorothioate (PTO)

## Authors' contributions

MW, CR and OK performed experiments and analyses. HH performed experiments and analyses and drafted the manuscript. DK conceived of the study, guided the practical work, data analyses and contributed to the writing of the manuscript. All authors read and approved of the final manuscript.

## Supplementary Material

Additional File 1 Number of clones received after transfection with different oligonucleotides. Table displaying the number of transfection experiments (with 10^6 ^transfected cells per experiment) per oligonucleotide and the number of clones received per experiment. The means and standard deviations of received clones and the number of clones sequenced per oligonucleotide are shown. Click here for file

## References

[B1] Andersen MS, Sorensen CB, Bolund L, Jensen TG (2002). Mechanisms underlying targeted gene correction using chimeric RNA/DNA and single-stranded DNA oligonucleotides. J Mol Med.

[B2] Kenner O, Kneisel A, Klingler J, Bartelt B, Speit G, Vogel W, Kaufmann D (2002). Targeted gene correction of hprt mutations by 45 base single-stranded oligonucleotides. Biochem Biophys Res Commun.

[B3] Liu L, Parekh-Olmedo H, Kmiec EB (2003). The development and regulation of gene repair. Nat Rev Genet.

[B4] Kmiec EB (1999). Targeted gene repair. Gene Ther.

[B5] Igoucheva O, Alexeev V, Yoon K (2004). Mechanism of gene repair open for discussion. Oligonucleotides.

[B6] Olsen PA, Randol M, Luna L, Brown T, Krauss S (2005). Genomic sequence correction by single-stranded DNA oligonucleotides: role of DNA synthesis and chemical modifications of the oligonucleotide ends. J Gene Med.

[B7] Parekh-Olmedo H, Ferrara L, Brachman E, Kmiec EB (2005). Gene therapy progress and prospects: targeted gene repair. Gene Ther.

[B8] Radecke F, Peter I, Radecke S, Gellhaus K, Schwarz K, Cathomen T (2006). Targeted Chromosomal Gene Modification in Human Cells by Single-Stranded Oligodeoxynucleotides in the Presence of a DNA Double-Strand Break. Mol Ther.

[B9] Igoucheva O, Alexeev V, Yoon K (2004). Oligonucleotide-directed mutagenesis and targeted gene correction: a mechanistic point of view. Curr Mol Med.

[B10] Huen MS, Lu LY, Liu DP, Huang JD (2007). Active transcription promotes single-stranded oligonucleotide mediated gene repair. Biochem Biophys Res Commun.

[B11] de Semir D, Aran JM (2006). Targeted gene repair: the ups and downs of a promising gene therapy approach. Curr Gene Ther.

[B12] Parekh-Olmedo H, Kmiec EB (2007). Progress and Prospects: targeted gene alteration (TGA). Gene Ther.

[B13] Liu L, Cheng S, van Brabant AJ, Kmiec EB (2002). Rad51p and Rad54p, but not Rad52p, elevate gene repair in Saccharomyces cerevisiae directed by modified single-stranded oligonucleotide vectors. Nucleic Acids Res.

[B14] Igoucheva O, Alexeev V, Yoon K (2002). Nuclear extracts promote gene correction and strand pairing of oligonucleotides to the homologous plasmid. Antisense Nucleic Acid Drug Dev.

[B15] Olsen PA, McKeen C, Krauss S (2003). Branched oligonucleotides induce in vivo gene conversion of a mutated EGFP reporter. Gene Ther.

[B16] Igoucheva O, Alexeev V, Scharer O, Yoon K (2006). Involvement of ERCC1/XPF and XPG in oligodeoxynucleotide-directed gene modification. Oligonucleotides.

[B17] Huen MS, Li XT, Lu LY, Watt RM, Liu DP, Huang JD (2006). The involvement of replication in single stranded oligonucleotide-mediated gene repair. Nucleic Acids Res.

[B18] Radecke F, Peter I, Radecke S, Gellhaus K, Schwarz K, Cathomen T (2006). Targeted Chromosomal Gene Modification in Human Cells by Single-Stranded Oligodeoxynucleotides in the Presence of a DNA Double-Strand Break. J Gene Med.

[B19] Maguire KK, Kmiec EB (2007). Multiple roles for MSH2 in the repair of a deletion mutation directed by modified single-stranded oligonucleotides. Gene.

[B20] Richardson PD, Kren BT, Steer CJ (2002). Gene repair in the new age of gene therapy. Hepatology.

[B21] Suzuki T, Murai A, Muramatsu T (2003). Low-dose bleomycin induces targeted gene repair frequency in cultured melan-c cells using chimeric RNA/DNA oligonucleotide transfection. Int J Mol Med.

[B22] Ferrara L, Kmiec EB (2004). Camptothecin enhances the frequency of oligonucleotide-directed gene repair in mammalian cells by inducing DNA damage and activating homologous recombination. Nucleic Acids Res.

[B23] Ferrara L, Parekh-Olmedo H, Kmiec EB (2004). Enhanced oligonucleotide-directed gene targeting in mammalian cells following treatment with DNA damaging agents. Cell Res.

[B24] Ferrara L, Kmiec EB (2006). Targeted gene repair activates Chk1 and Chk2 and stalls replication in corrected cells. DNA Repair (Amst).

[B25] Schwartz TR, Kmiec EB (2007). Reduction of gene repair by selenomethionine with the use of single-stranded oligonucleotides. BMC Mol Biol.

[B26] Ferrara L, Engstrom JU, Schwartz T, Parekh-Olmedo H, Kmiec EB (2007). Recovery of cell cycle delay following targeted gene repair by oligonucleotides. DNA Repair (Amst).

[B27] Kenner O, Kneisel A, Klingler J, Bartelt B, Speit G, Vogel W, Kaufmann D (2002). Targeted gene correction of hprt mutations by 45 base single-stranded oligonucleotides. Biochem Biophys Res Commun.

[B28] Agarwal S, Gamper HB, Kmiec EB (2003). Nucleotide replacement at two sites can be directed by modified single-stranded oligonucleotides in vitro and in vivo. Biomol Eng.

[B29] Yin WX, Wu XS, Liu G, Li ZH, Watt RM, Huang JD, Liu DP, Liang CC (2005). Targeted correction of a chromosomal point mutation by modified single-stranded oligonucleotides in a GFP recovery system. Biochem Biophys Res Commun.

[B30] Mu D, Hsu DS, Sancar A (1996). Reaction mechanism of human DNA repair excision nuclease. J Biol Chem.

[B31] Sijbers AM, de Laat WL, Ariza RR, Biggerstaff M, Wei YF, Moggs JG, Carter KC, Shell BK, Evans E, de Jong MC, Rademakers S, de Rooij J, Jaspers NG, Hoeijmakers JH, Wood RD (1996). Xeroderma pigmentosum group F caused by a defect in a structure-specific DNA repair endonuclease. Cell.

[B32] Prasad R, Lavrik OI, Kim SJ, Kedar P, Yang XP, Vande Berg BJ, Wilson SH (2001). DNA polymerase beta-mediated long patch base excision repair. Poly(ADP-ribose) polymerase-1 stimulates strand displacement DNA synthesis. J Biol Chem.

[B33] Christmann M, Tomicic MT, Roos WP, Kaina B (2003). Mechanisms of human DNA repair: an update. Toxicology.

[B34] Aarts M, Dekker M, de Vries S, van der Wal A, te Riele H (2006). Generation of a mouse mutant by oligonucleotide-mediated gene modification in ES cells. Nucleic Acids Res.

[B35] Rice MC, Bruner M, Czymmek K, Kmiec EB (2001). In vitro and in vivo nucleotide exchange directed by chimeric RNA/DNA oligonucleotides in Saccharomyces cerevisae. Mol Microbiol.

[B36] Dekker M, Brouwers C, Aarts M, van der Torre J, de Vries S, van de Vrugt H, te Riele H (2006). Effective oligonucleotide-mediated gene disruption in ES cells lacking the mismatch repair protein MSH3. Gene Ther.

[B37] Ellis HM, Yu D, DiTizio T, Court DL (2001). High efficiency mutagenesis, repair, and engineering of chromosomal DNA using single-stranded oligonucleotides. Proc Natl Acad Sci U S A.

[B38] Dekker M, Brouwers C, te Riele H (2003). Targeted gene modification in mismatch-repair-deficient embryonic stem cells by single-stranded DNA oligonucleotides. Nucleic Acids Res.

[B39] Brachman EE, Kmiec EB (2004). DNA replication and transcription direct a DNA strand bias in the process of targeted gene repair in mammalian cells. J Cell Sci.

[B40] Wu XS, Xin L, Yin WX, Shang XY, Lu L, Watt RM, Cheah KS, Huang JD, Liu DP, Liang CC (2005). Increased efficiency of oligonucleotide-mediated gene repair through slowing replication fork progression. Proc Natl Acad Sci U S A.

[B41] Olsen PA, Randol M, Krauss S (2005). Implications of cell cycle progression on functional sequence correction by short single-stranded DNA oligonucleotides. Gene Ther.

[B42] Blumenthal AB, Clark EJ (1977). Discrete sizes of replication intermediates in Drosophila cells. Cell.

[B43] Bielinsky AK, Gerbi SA (1999). Chromosomal ARS1 has a single leading strand start site. Mol Cell.

[B44] Zerbe LK, Kuchta RD (2002). The p58 subunit of human DNA primase is important for primer initiation, elongation, and counting. Biochemistry.

[B45] Gamper HB, Parekh H, Rice MC, Bruner M, Youkey H, Kmiec EB (2000). The DNA strand of chimeric RNA/DNA oligonucleotides can direct gene repair/conversion activity in mammalian and plant cell-free extracts. Nucleic Acids Res.

